# Association of self-efficacy, risk attitudes, and time preferences with functioning in older patients with vertigo, dizziness, and balance disorders in a tertiary care setting—Results from the MobilE-TRA2 cohort

**DOI:** 10.3389/fneur.2023.1316081

**Published:** 2023-12-15

**Authors:** Benedict Katzenberger, Sebastian Fuchs, Lars Schwettmann, Ralf Strobl, Ari Hauser, Daniela Koller, Eva Grill

**Affiliations:** ^1^Institute for Medical Information Processing, Biometry and Epidemiology (IBE), Faculty of Medicine, LMU Munich, Munich, Germany; ^2^Pettenkofer School of Public Health, Munich, Germany; ^3^Department of Orthopaedics and Trauma Surgery, LMU University Hospital, LMU Munich, Munich, Germany; ^4^Department of Health Services Research, School of Medicine and Health Sciences, Carl Von Ossietzky University of Oldenburg, Oldenburg, Germany; ^5^Institute of Health Economics and Health Care Management (IGM), Helmholtz Zentrum München (GmbH) – German Research Center for Environmental Health, Neuherberg, Germany; ^6^German Center for Vertigo and Balance Disorders, LMU University Hospital, LMU Munich, Munich, Germany

**Keywords:** vertigo, dizziness, balance disorders, functioning, self-efficacy, risk attitudes, time preferences

## Abstract

**Introduction:**

The functional burden of vertigo, dizziness, and balance problems (VDB) might depend on the personality traits of the patients affected. The aim of this study thus was to investigate the impact of self-efficacy, risk attitudes, and time preferences on functioning in older patients with VDB before and after treatment in a specialized tertiary care center.

**Methods:**

Data for this study was obtained from the MobilE-TRA2 cohort study, conducted at a specialized tertiary care center in Germany. Patients aged 60 and older were assessed during their initial stay at the care center and 3 months later, using self-administered questionnaires. Self-efficacy was measured on a scale from 1 (very low) to 5 (very high). Health-related risk attitudes were inquired using an 11-point scale. Time preferences were measured by evaluating patients' willingness to postpone a reward in favor of a greater benefit on an 11-point Likert scale. Functioning was evaluated using the Dizziness Handicap Inventory, representing functional, emotional, and physical aspects of functional disability caused by VDB. Mixed-effects regression models were used to analyze the association between the selected personality traits and functioning over time. Interaction terms with time were incorporated for each personality trait, enabling the assessment of their influence on functioning 3 months following the initial observation period.

**Results:**

An overall of 337 patients (53% women, median age at baseline = 70 years) were included. Patients with higher self-efficacy (Beta = −3.82, 95%-CI [−6.56; −1.08]) and higher willingness to take risks (Beta = −1.31, 95%-CI [−2.31; −0.31]) reported better functioning during their initial visit at the care center. Self-efficacy significantly predicted functioning after 3 months for overall functioning (Beta = −4.21, 95%-CI [−6.57; −1.84]) and all three domains.

**Conclusion:**

Our findings suggest that patients with high self-efficacy and high willingness to take risks may exhibit better coping mechanisms when faced with the challenges of VDB. Promoting self-efficacy may help patients to better manage the duties accompanying their treatment, leading to improved functioning. These insights may inform the development of personalized treatment aimed at reducing the functional burden of VDB in older patients.

## 1 Introduction

Vertigo, dizziness, and balance problems (VDB) are common and challenging syndromes, especially in older adults. They affect over 30% of the population over the age of 60 (1–3) and result in relevant and often persistent functional decline (4). While VDB commonly are a result of disorders of the vestibular system, they also can be provoked by other diseases, such as orthostatic hypotension or polyneuropathy. People with VDB are often faced with a wide range of problems in carrying out tasks of their daily lives (5, 6), including work-related and social activities (7). Household chores and grocery shopping, traveling, reading, and even walking, bending over or dressing can become challenging (8, 9). Depression and anxiety are common comorbidities (10, 11), arguably provoked by feelings of reduced self-esteem, fear, vulnerability, frustration, and isolation (12).

Earlier research suggests that people with certain personality structures are particularly susceptible to VDB and its effects (13, 14) and that individuals differ in their ways of coping with functional restrictions caused by VDB (15). In the context of highly specialized tertiary care, personality traits might influence the patients' ability to navigate the challenges inherent in their care process, as observed in the management of chronic diseases (16). As such, it is conceivable that personality traits may hold predictive value in determining treatment outcomes to some extent. Nevertheless, the current state of research on the impact of personality traits on functional restrictions in patients with VDB remains sparse.

Further insights may come from the field of behavioral economics, which is a branch of economics that combines approaches and methods from economics and psychology to understand how and why individuals make decisions and choices (17). Concepts from behavioral economics recognize that patients may not consistently conform to the expected rationality when coping with their disease and the associated restrictions. This deviation can be attributed to variations in underlying personality traits, including but not limited to the patient's confidence in their ability to overcome a health problem, their risk-taking propensity, and the way they assess the benefits or harms that lie in the distant future (18). Such insights could be helpful to better understand and predict functional restrictions in patients with VDB. The present work focuses on three prominent personality traits, namely self-efficacy, individual risk attitudes, and time preferences, as three selected BE concepts in the realm of health-related decision-making, which are known to be major determinants of health behavior (16, 19–21).

Self-efficacy denotes the belief in one's ability to organize and execute the courses of action required to successfully achieve set goals (22). The positive influence of self-efficacy on various health outcomes is well-known in the literature (16, 23–25). With regard to VDB, it may indicate patients' willingness to actively confront their problems. This has been shown, e.g., for visual height intolerance (26), a condition characterized by discomfort or anxiety when individuals are exposed to heights or elevated places, even if they are safe and enclosed. Likewise, patients with high levels of self-efficacy and resilience were less likely to develop secondary somatoform dizziness and vertigo (27). Also, internal health locus of control, i.e., the belief that individuals themselves are in control of managing their health condition (28), was found to support coping in VDB (29). Although internal locus of control encompasses a broader belief in the control over one's health condition, while self-efficacy is more focused on task-specific confidence, these findings underline the importance of patients' perceptions of control.

Health-related risk attitudes refer to an individual's general propensity to take or avoid risks in health-related decision situations (21). They hold significance in understanding coping styles within VDB, where individuals regularly face risk-related decisions linked to daily activities, involving certain actions or environments that may exacerbate symptoms. Moreover, individuals with VDB have to consider their elevated propensity for falls (30, 31). Patients with VDB thus must carefully assess the level of engagement in health-related activities, such as performing physical exercise, that pose some risk of triggering symptoms or falls but may contribute to maintaining overall functioning. Some individuals may be very risk-averse, resulting in excessive caution (11, 32), exaggerated self-restriction, or even complete avoidance of environments they perceive as safe (33). Such self-imposed restriction could then result in adverse consequences, including diminished core stability and restricted participation in various activities. Conversely, those more willing to take risks may not be as susceptible to fear and exaggerated self-restriction possibly mitigating the negative impact of VDB on functioning.

Finally, time preferences, reflecting the patient's valuation of the present over the future when deciding between immediate health benefits or harms and potential benefits or harms in the future (34–36), can influence health behavior. It has been demonstrated that more present-oriented individuals tend to be less likely to adopt healthy lifestyles than future-oriented individuals (20, 37) and engage in fewer self-management activities (38, 39). On the other hand, individuals with a stronger present orientation reported lower levels of concern about future illness (40). Consequently, they might be more optimistic about the future and therefore experience lower levels of self-imposed restrictions (33) than their future-oriented counterparts.

It is widely recognized that patients with VDB derive substantial benefit from evidence-based and interdisciplinary rehabilitation programs in many different underlying pathologies (41–45). The effects of such rehabilitation programs seem to be even larger when the interventions are tailored to the patients' specific needs (46). In this context, gaining a better understanding of how the selected personality traits influence functioning and recovery in patients with VDB before and after their visit to a highly specialized tertiary care center may help to adapt and further improve existing therapeutic approaches.

The objective of this article thus was to investigate the impact of self-efficacy, risk attitudes, and time preferences on the development of functioning in older patients with VDB before and after treatment in a specialized tertiary care setting.

## 2 Materials and methods

### 2.1 Study design and study population

Data for this research project was collected in the prospective cohort study MobilE-TRA2 (“Behavioral and patient-individual determinants of quality of life, functioning and physical activity in older adults”) at the interdisciplinary outpatient clinic of the German Center for Vertigo and Balance Disorders (DSGZ) at the Munich University Hospital. The DSGZ is one of the world's leading centers for diagnosis, treatment, and research of vertigo. Patients usually present at the clinic after referral from primary care. The study included patients aged 60 and older with VDB who presented for their initial interdisciplinary evaluation at the DSGZ. Patients with terminal diseases, cognitive impairment, or insufficient command of the German language were excluded. A more detailed description of the study is given elsewhere (47).

The sample size calculation for MobilE-TRA 2 was guided by a clinically significant difference of 10.0 points on the DHI, assuming a standard deviation of 25. Targeting a power of 0.8 (alpha = 0.05) necessitated a sample size of 52 patients. Given the longitudinal nature of MobilE-TRA 2, spanning three waves, and anticipating a 20% loss to follow-up between each wave, we established a minimum sample size of 81 patients. As different underlying pathologies had to be considered in order to control for their impact on our estimates, we quadrupled this figure, arriving at a target sample size of 324 patients.

The MobilE-TRA-2 study received ethics approval from the Ethics Committee at the medical faculty of Ludwig Maximilian University of Munich (#20-727). Written informed consent was obtained from all participants and the study was performed in accordance with the Declaration of Helsinki principles.

### 2.2 Data collection procedures

Baseline assessment was conducted between December 2020 and June 2022 and consisted of a self-administered questionnaire which patients either completed during their stay at the DSGZ or sent back via postal mail. Information from the patient registry DizzyReg of the DSGZ (48) was used to complement the baseline assessment. In brief, DizzyReg is an ongoing prospective clinical patient registry that collects and combines information stored in electronic health records and medical discharge letters with patient-reported information gathered by self-administered questionnaires. For the follow-up of the MobilE-TRA2 cohort, patients were mailed a questionnaire 3 months after the individual baseline assessment. Patients who did not respond to the initial follow-up questionnaire within 1 month received a reminder and were supplied with an identical duplicate of the initial follow-up questionnaire.

### 2.3 Personality traits

Self-efficacy was rated based on the three items of the General Self-Efficacy Short Scale (49). Patients report their confidence that they (1) can rely on their abilities in difficult situations, (2) can handle most problems well on their own, and (3) can usually solve even demanding and complex tasks effectively. The level of confidence is rated on a scale from 1 (“doesn't apply at all”) to 5 (“applies completely”). The level of self-efficacy was calculated as the arithmetic mean of all three answers, resulting in a scale from 1 (very low self-efficacy) to 5 (very high self-efficacy). To measure health-related risk attitudes, a single item with an 11-point scale was used, ranging from 0 (“not at all willing to take risks”) to 10 (“very willing to take risks”) (21). The concept of time preferences used in this analysis was assessed by two items. One item measures the willingness to postpone a reward (0 = not willing at all, 10 = very willing) for the sake of a greater benefit in the future (36) and one item assesses the patient's orientation toward the present rather than in the future (“I am only concerned about the present, because I trust that things will work themselves out in the future,” 1 = totally disagree, 5 = “totally agree”) (34).

### 2.4 Functioning

Functioning was assessed using the German version of the Dizziness Handicap Inventory (DHI) (8, 50). The DHI is the most commonly used instrument to assess functioning loss caused by dizziness in everyday activities, including activity limitation, participation restrictions, and experienced difficulties. It incorporates 25 single items that can be summarized into three domains, representing functional (range 0–36), physical (range 0–28), and emotional (range 0 – 36) aspects of functioning, as well as a total score (range 0–100). Higher scores indicate more severe limitation or restriction.

### 2.5 Covariables

The selection of covariables for this study was based on the directed acyclic graph (DAG) presented in Supplementary Figure S1. This approach allowed us to identify the minimal sufficient adjustment set necessary to control for potential confounding while simultaneously avoiding bias from over-adjustment or collider bias (51). The construction of the DAG was informed by a review of the literature and experts' knowledge at the DSGZ. The resulting minimal adjustment set contained the specific VDB diagnosis, multimorbidity, a history of falls prior to the visit at the DSGZ, as well as information on the age, gender, education, and marital status of the participants.

The specific diagnosis of VDB was based on an extensive neurootological workup performed at the DSGZ, conforming to current guidelines (52–57). This workup includes a comprehensive battery of bedside tests, audiologic and vestibular function tests, as well as imaging if necessary. We focused on the most frequent diagnoses at the DSGZ, namely benign paroxysmal positional vertigo (BPPV), unilateral vestibulopathy, bilateral vestibulopathy, Menière's disease, vestibular migraine, central vestibular disorders, functional vertigo, orthostatic vertigo, and vertigo caused by polyneuropathy. Less frequent diagnoses were assigned to “Other” to facilitate statistical analysis. If no single leading cause was identifiable by the experts at the DSGZ, patients were classified as having multifactorial VDB.

Additional information was provided by patients regarding existing comorbidities related to the heart, lungs, liver, kidneys, neurological conditions, high blood pressure, inflammatory joint diseases, and further diseases specifically indicated by the participants. This approach yielded a compilation of 13 potential comorbidities, which can be found in Supplementary Table S1. We used this information to identify multimorbid patients, i.e., patients that suffered from at least two chronic conditions in addition to VDB. Multi-morbidity was added as binary information (yes/no) in the analysis.

During baseline assessment, patients reported whether they had fallen within the last 12 months prior to their visit at the DSGZ using a single yes-or-no question. Information on age and gender (male/female) was based on patients' self-report. Education levels were categorized based on the German educational system into: no graduation, lower secondary education 1 (equals 9 years of school), lower secondary education 2 (equals 10 years of school), upper secondary education (equals 12 or 13 years of school), and tertiary education (university, university of applied sciences). Marital status was self-reported (single, married, divorced, or widowed).

### 2.6 Statistical analysis

Summary statistics were calculated for the overall sample and separately for each diagnosis of VDB. Mean and standard deviation were reported for normally distributed continuous variables, median and the interquartile range for non-normally distributed variables, and relative and absolute frequencies for categorical variables. Potential differences in the observed variables between different diagnoses of VDB were examined using one-way ANOVA for normally distributed continuous variables, Kruskal-Wallis test for non-normally distributed continuous variables, and Chi-squared test for categorical variables.

Longitudinal linear mixed models with random intercept and fixed slopes were applied to assess the association between the selected personality traits and the level of functioning over time. We computed four distinct models: one for the overall DHI to estimate overall functioning and separate models for each of the three DHI sub-scales. Each beta coefficient obtained from the models represents the estimated change in the respective DHI score associated with a one-unit change in the corresponding predictor variable while controlling for the influence of all other variables in the model. Within each model, we simultaneously integrated the variables indicating self-efficacy, risk attitudes, and time preferences. This approach allowed us to accurately estimate the impact of each personality trait while simultaneously controlling for the influence of the other two traits. To assess potential multicollinearity issues among the personality traits and other covariates, we computed variance inflation factors (VIF) (58) using a predetermined threshold of 5 points. Furthermore, we introduced interaction terms involving time for each personality trait within each model, enabling us to investigate whether changes in functioning over time were predicted by the patients' respective personality traits.

In the regression analyses, we adopted a strategy of centering the measures of self-efficacy, risk attitudes, and time preferences around their respective means. This decision was informed by the observed concentration of values around the mid-range, with comparatively few instances of extremely low or high values. By employing centered models, we derived estimators for the intercept and overall change over time that are representative for individuals with moderate levels of these personality traits. These estimators directly capture a significantly larger portion of our study cohort compared to non-centered models. Given the minimum age criterion established in the inclusion criteria, we also subtracted the minimum age of 60 from the patients' age in years. Consequently, the reported estimates for the intercepts and the overall change over time in the centered models apply to patients at the age of 60 with mean personality traits.

Time preferences were represented by patients' willingness to postpone a reward within the primary analysis. To assess the robustness of our findings, we performed sensitivity analyses in which we re-evaluated the identical longitudinal linear mixed models. However, in these analyses, we measured time preferences based on patients' present-time orientation (“I am only concerned about the present, because I trust that things will work themselves out in the future”). This was done to examine whether the specific assessment of time preferences has an impact on the results.

The significance level was set to 5%. All computational analyses were carried out with R Version 4.1.2, including the usage of the *nlme* library (59). Regression assumptions were tested visually. We employed DAGitty, a browser-based, open-source tool to construct, edit, and analyze the DAG central to our study (60). In essence, users utilize a graphical interface to create the DAG, and the tool automatically identifies and highlights causal and biasing paths using distinct colors. This dynamic feature allows researchers to promptly and interactively assess the impact of DAG modifications, such as adding new arrows or variables or inverting arrows with unclear causal direction. Additionally, DAGitty identifies the minimal adjustment set by and underlying algorithm, providing real-time feedback to the user along with the underlying assumptions.

## 3 Results

A total of 337 patients (53 % women, median age at baseline = 70 years, IQR = [64, 78]) were included in the baseline assessment. Of these, 299 (89%) returned the follow-up questionnaires, which were sent out 3 months after their respective baseline assessment. The most frequent diagnoses at baseline were BPPV [*n* = 48, (21%)] and functional vertigo (*n* = 48), followed by balance problems caused by polyneuropathy (*n* = 43). Thirty patients were classified as having multifactorial VDB. A third (*n* = 112, 33%) of the patients reported to have experienced at least one fall within the last 12 months prior to their visit. Additional details can be found in Table 1.

**Table 1:** Unadjusted summary statistics stratified by diagnosis at baseline assessment (*n* = 337).

	**Diagnosis**												
	**Overall**	**BPPV**	**Unilateral vestibulopathy**	**Bilateral vestibulopathy**	**Ménière'sdisease**	**Vestibular migraine**	**Centralvestibular**	**Functional vertigo**	**Orthostatic vertigo**	**Polyneuropathy**	**Multifactorial**	**Other**	***p*** **-value** ^a^
*N* (%)	337	48	18	14	33	29	22	48	29	43	30	23	
**Socio-economic information and medical background**													
Age (median, IQR)	70.00 [64.00, 78.00]	69.00 [65.00, 77.25]	67.00 [60.50, 70.50]	69.00 [64.75, 77.75]	72.00 [67.00, 77.00]	64.00 [61.00, 71.00]	68.50 [63.00, 75.75]	64.50 [61.75, 70.25]	75.00 [62.00, 78.00]	78.00 [73.00, 81.50]	77.50 [73.25, 82.00]	65.00 [62.50, 71.50]	<0.001
Gender (*n*, %)													
Female	179 (53.1)	29 (60.4)	8 (44.4)	6 (42.9)	21 (63.6)	20 (69.0)	9 (40.9)	39 (81.2)	13 (44.8)	15 (34.9)	11 (36.7)	8 (34.8)	<0.001
Male	158 (46.9)	19 (39.6)	10 (55.6)	8 (57.1)	12 (36.4)	9 (31.0)	13 (59.1)	9 (18.8)	16 (55.2)	28 (65.1)	19 (63.3)	15 (65.2)	
Fall within last 12 months (*n*, %)													
No	220 (66.3)	30 (63.8)	12 (66.7)	8 (57.1)	26 (78.8)	25 (86.2)	14 (66.7)	34 (72.3)	16 (55.2)	26 (61.9)	16 (53.3)	13 (59.1)	0.192
Yes	112 (33.7)	17 (36.2)	6 (33.3)	6 (42.9)	7 (21.2)	4 (13.8)	7 (33.3)	13 (27.7)	13 (44.8)	16 (38.1)	14 (46.7)	9 (40.9)	
Multimorbidity (≥2 Comorbidities) (*n*, %)													
No	142 (42.1)	16 (33.3)	8 (44.4)	6 (42.9)	19 (57.6)	13 (44.8)	10 (45.5)	23 (47.9)	12 (41.4)	12 (27.9)	9 (30.0)	14 (60.9)	0.158
Yes	195 (57.9)	32 (66.7)	10 (55.6)	8 (57.1)	14 (42.4)	16 (55.2)	12 (54.5)	25 (52.1)	17 (58.6)	31 (72.1)	21 (70.0)	9 (39.1)	
Education^b^ (*n*, %)													
Lower secondary education 1	119 (36.3)	18 (37.5)	6 (35.3)	5 (35.7)	12 (37.5)	9 (32.1)	6 (27.3)	13 (28.3)	14 (51.9)	17 (39.5)	12 (41.4)	7 (31.8)	0.981
Lower secondary education 2	81 (24.7)	14 (29.2)	5 (29.4)	4 (28.6)	5 (15.6)	5 (17.9)	10 (45.5)	14 (30.4)	4 (14.8)	10 (23.3)	6 (20.7)	4 (18.2)	
Upper secondary education	40 (12.2)	4 (8.3)	2 (11.8)	2 (14.3)	5 (15.6)	5 (17.9)	2 (9.1)	6 (13.0)	3 (11.1)	6 (14.0)	2 (6.9)	3 (13.6)	
Tertiary education	87 (26.5)	11 (22.9)	4 (23.5)	3 (21.4)	10 (31.2)	9 (32.1)	4 (18.2)	13 (28.3)	6 (22.2)	10 (23.3)	9 (31.0)	8 (36.4)	
No graduation	1 (0.3)	1 (2.1)	0 (0.0)	0 (0.0)	0 (0.0)	0 (0.0)	0 (0.0)	0 (0.0)	0 (0.0)	0 (0.0)	0 (0.0)	0 (0.0)	
Marital status (*n*, %)													
Single	22 (6.5)	6 (12.5)	3 (16.7)	1 (7.1)	1 (3.0)	1 (3.4)	2 (9.1)	2 (4.2)	3 (10.3)	0 (0.0)	2 (6.7)	1 (4.3)	0.727
Married	227 (67.4)	31 (64.6)	13 (72.2)	9 (64.3)	22 (66.7)	18 (62.1)	17 (77.3)	33 (68.8)	20 (69.0)	28 (65.1)	20 (66.7)	16 (69.6)	
Divorced	49 (14.5)	7 (14.6)	2 (11.1)	2 (14.3)	4 (12.1)	6 (20.7)	3 (13.6)	9 (18.8)	2 (6.9)	6 (14.0)	4 (13.3)	4 (17.4)	
Widowed	39 (11.6)	4 (8.3)	0 (0.0)	2 (14.3)	6 (18.2)	4 (13.8)	0 (0.0)	4 (8.3)	4 (13.8)	9 (20.9)	4 (13.3)	2 (8.7)	
**Personality traits**													
Self-efficacy (mean, SD)	3.96 (0.85)	3.85 (0.74)	4.02 (0.77)	4.03 (0.75)	4.00 (0.69)	4.25 (0.89)	3.83 (0.91)	4.02 (0.93)	4.00 (1.10)	3.75 (0.85)	3.94 (0.74)	4.03 (1.01)	0.562
Health-related risk attitudes (mean, SD)	4.63 (2.33)	4.34 (2.42)	4.59 (2.58)	5.21 (2.58)	5.36 (2.25)	3.68 (2.13)	4.59 (2.61)	4.19 (2.21)	5.14 (2.22)	4.76 (2.01)	4.80 (2.12)	4.83 (2.82)	0.217
Time preferences—willingness to postpone a reward (mean, SD)	6.19 (2.16)	6.15 (2.04)	7.50 (1.95)	6.79 (2.26)	7.03 (2.01)	6.44 (2.34)	5.86 (2.05)	5.42 (2.19)	5.86 (2.21)	5.62 (1.93)	6.50 (1.85)	6.39 (2.48)	0.007
Time preferences—present-orientation (mean, SD)	2.53 (1.03)	2.37 (0.97)	2.41 (1.06)	2.57 (1.09)	2.68 (1.08)	2.42 (1.06)	2.62 (0.86)	2.71 (0.92)	2.82 (1.09)	2.54 (1.10)	2.34 (1.11)	2.18 (1.10)	0.490
**Functioning**													
DHI overall score (mean, SD)	40.93 (21.40)	43.83 (22.39)	45.00 (23.09)	48.57 (20.58)	43.67 (19.92)	32.83 (24.41)	44.00 (22.35)	47.70 (17.87)	29.21 (14.89)	38.70 (21.05)	44.64 (20.87)	28.10 (21.59)	0.001
DHI functional score (mean, SD)	16.53 (9.80)	17.17 (10.50)	17.75 (9.49)	19.29 (8.69)	17.74 (8.34)	13.23 (10.73)	18.00 (10.32)	19.62 (8.99)	13.21 (7.88)	15.35 (9.08)	18.50 (10.99)	10.19 (9.59)	0.007
DHI physical score (mean, SD)	11.41 (6.95)	13.87 (6.03)	14.25 (7.41)	15.00 (7.72)	10.13 (7.78)	7.67 (6.66)	12.57 (6.85)	11.67 (6.34)	7.50 (4.95)	11.68 (7.98)	12.69 (5.81)	9.00 (6.26)	<0.001
DHI emotional score (mean, SD)	13.04 (8.14)	12.78 (8.78)	13.00 (8.36)	14.29 (7.68)	16.00 (8.27)	11.62 (8.73)	13.43 (8.51)	16.38 (6.66)	8.50 (6.41)	11.68 (7.17)	13.50 (8.28)	10.27 (8.93)	0.003

BPPV, Benign paroxysmal positional vertigo; DHI, Dizziness Handicap Inventory; IQR, Interquartile range; SD, Standard deviation. ^a^ANOVA, Kruskal-Wallis test, Chi-squared test. ^b^Lower secondary education 1 equals 9 years of school, Lower secondary education 2 equals 10 years of school, upper secondary education equals 12 or 13 years of school.

The mean overall DHI score across all patients at baseline was 41 points, indicating a moderate level of handicap due to VDB, with 62 (18%) patients reaching an overall DHI of more than 60 points (severe handicap) (61). The overall DHI score as well as all three sub-scores differed significantly across the diagnoses of VDB. Patients with bilateral vestibulopathy and patients with functional vertigo reported the highest level of impairment with DHI scores of 48.57 and 47.70, respectively. Conversely, patients with orthostatic vertigo and patients with other forms of VDB presented with better functioning (DHI = 29.21, resp. DHI = 28.10). The patients' willingness to postpone a reward (mean = 6.19, SD = 2.16) differed significantly across VDB diagnoses. Patients with functional vertigo exhibited the lowest willingness to delay gratification (mean = 5.42, SD = 2.19). The measures related to self-efficacy (mean = 3.96, SD = 0.85) and willingness to take risks (mean = 4.63, SD = 2.33) did not significantly differ across the various VDB diagnoses.

The mean overall DHI score at the follow-up assessment was 39 points across all patients. The overall DHI score again varied across the diagnoses of VDB, with corresponding differences in the functional and physical sub-scales of the DHI. A comprehensive list of the overall DHI scores and the three distinct DHI sub-scales for each diagnosis at follow-up is provided in Supplementary Table S2.

Adjusted for all covariates, the overall functional status increased on average by 2.56 points (95%-CI [−4.47; −0.65]) over the course of 3 months. Patients with higher self-efficacy reported better overall functioning at baseline (Beta = −3.82, 95%-CI [−6.56; −1.08]) and experienced greater improvement after 3 months (Beta = −4.21, 95%-CI [−6.57; −1.84]). While patients displaying a greater willingness to take risks reported slightly better overall functioning at baseline (Beta = −1.31, 95%-CI [−2.31; −0.31]), there were no statistically significant differences in their rate of improvement over time compared to risk-averse patients. Time preferences were neither significantly associated with baseline functioning nor with improvement over time. More detailed results of the mixed models for the DHI overall scales and the DHI subscales are presented in Table 2.

**Table 2:** Longitudinal linear mixed models to assess the association between personality traits and functioning.

	**Dizziness handicap inventory (95%–CI)**			
	**M1: overall score**	**M2: functional score**	**M3: physical score**	**M4: emotional score**
Observations (n)	557 (305)	559 (305)	560 (306)	562 (306)
(Intercept)^a^	44.53 (33.07; 56.00)	18.38 (13.13; 23.62)	11.75 (7.95; 15.55)	14.49 (10.20; 18.79)
**Wave** ^a^				
Baseline	Reference	Reference	Reference	Reference
Follow-up (3 months later)	**−2.56 (−4.47;** **−0.65)**	**−1.17 (−2.03;** **−0.32)**	−0.04 (−0.75; 0.67)	**−1.44 (−2.17;** **−0.71)**
**Personality traits (centered to the respective mean)**				
**Self-efficacy** ^b^				
Self-efficacy	**−3.82 (−6.56;** **−1.08)**	**−1.76 (−3.01;** **−0.52)**	−0.60 (−1.51; 0.31)	**−1.49 (−2.51;** **−0.48)**
Self-efficacy^*^ time	**−4.21 (−6.57;** **−1.84)**	**−1.65 (−2.71;** **−0.59)**	**−1.03 (−1.89;** **−0.17)**	**−1.43 (−2.32;** **−0.55)**
**Health-related risk attitudes** ^c^				
Risk attitudes	**−1.31 (−2.31;** **−0.31)**	**−0.54 (−0.99;** **−0.09)**	−0.07 (−0.40; 0.27)	**−0.71 (−1.09;** **−0.34)**
Risk attitudes^*^ time	0.29 (−0.56; 1.14)	0.16 (−0.22; 0.54)	−0.01 (−0.32; 0.31)	0.13 (−0.20; 0.45)
**Time preferences—willingness to postpone a reward (WPR)** ^d^				
WPR	−0.38 (−1.48; 0.73)	−0.21 (−0.72; 0.29)	0.11 (−0.25; 0.48)	−0.28 (−0.69; 0.13)
WPR^*^ time	−0.12 (−1.05; 0.81)	0.03 (−0.39; 0.44)	−0.21 (−0.55; 0.13)	0.01 (−0.34; 0.36)
**Random effects**				
Intercept (SD)	15.49	7.13	4.89	5.75

Higher scores indicate worse functioning. Significant findings are printed in bold. All models are controlled for the diagnosis, present falls within the last 12 months, multimorbidity, age, gender, education, and marital status. CI, Confidence interval; M1–M4, Models 1 to 4, one model per score; SD, Standard deviation. ^a^Applies for patients aged 60 with mean self-efficacy of 3.96, mean risk attitudes of 4.63, and mean willingness to postpone a reward of 6.19. ^b^Centered to mean self-efficacy of 3.96. ^c^Centered to mean risk attitudes of 4.63. ^d^Centered to mean willingness to postpone a reward of 6.19.

The performed sensitivity analysis revealed that using the patient's orientation toward the present as an alternative operationalization of time preferences did not change the association found in the main model (Supplementary Table S3). This consistency underscores the robustness of our findings.

**Figure 1:**
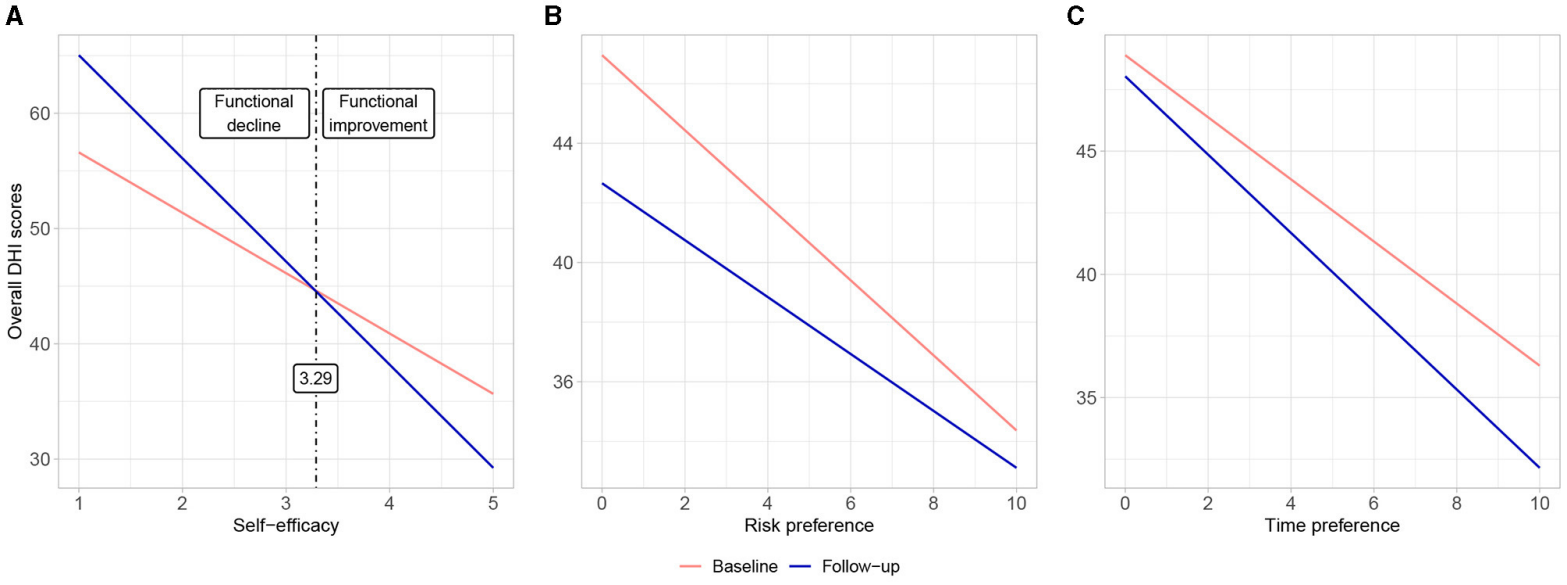
Comparison of predicted values for the overall functioning during baseline and follow-up assessment (3 months later) for different values of **(A)** self-efficacy, **(B)** risk attitudes, and **(C)** time preferences (willingness to postpone a reward). Higher values in the DHI scores indicate worse functioning.

Figure 1 displays a graphical representation of the predicted values from our models. It illustrates the overall functioning at the baseline assessment and the three-month follow-up for various values of the selected personality traits. Based on these predictions, a threshold of 3.29 points on the self-efficacy scale was identified as necessary for patients to experience an improvement in functioning over the course of 3 months. Patients below this threshold had lower functioning compared to their baseline assessment.

## 4 Discussion

We analyzed the impact of self-efficacy, risk attitudes, and time preferences on functioning in older patients with vertigo, dizziness, and balance disorders (VDB) using cohort data from a specialized tertiary academic care clinic. Patients with higher self-efficacy and more willingness to take risks reported higher levels of functioning when presenting at the clinic. Higher self-efficacy was also found to be an independent predictor of a better recovery at 3 months after initial assessment.

Overall, patients showed a small but significant improvement in functioning over time which might in part be due to the standardized diagnostic workup at the DSGZ and the long-standing experience of the clinic with management of VDB. These findings are in good agreement with previous research, highlighting the potential benefits of evidence-based and interdisciplinary assessment and vestibular rehabilitation therapy (42, 44) for patients with VDB.

Our analysis showed that patients with higher levels of self-efficacy were less restricted by their symptoms. These findings align with previous studies that have emphasized the positive influence of self-efficacy on various health outcomes, both within VDB (26, 27) and in general (16, 23, 25). Our results suggest that individuals who have a greater belief in their abilities to handle difficult situations, solve problems, and rely on their skills might have already developed effective coping mechanisms and adopted them in their daily life, thereby mitigating the impact of VDB on their functioning.

Our most striking observation was that self-efficacy predicted functional status of the patients 3 months after their initial visit. This indicates that patients may require a certain level of self-efficacy to experience functional improvement over time. Patients with very high self-efficacy demonstrated remarkable improvements, while those with low self-efficacy displayed an even lower functioning status after 3 months than during their first visit at the DSGZ. This suggests that self-efficacy may have a dual role in VDB diagnostics, serving as both a prerequisite and a catalyst for functional improvement after visiting a specialized care center. One possible explanation for this observation lies in the challenges that patients may find themselves confronted with when leaving the care center after their initial visit. Recommendations for future treatment options which, depending on the underlying pathology, may involve exercises, consultations with specialists, or additional diagnostic procedures, often require patients to take on new responsibilities and learn new skills. Consistent with this, previous research on self-efficacy in the management of chronic diseases suggests that patients with high self-efficacy are better able to cope with these challenges (16). This likely applies to patients with VDB leaving the care center as well. Therefore, empowering patients to develop confidence in their abilities to actively participate in their treatment should be an important pillar of future treatment strategies. Self-efficacy enhancing interventions, which have proven to be of use in many other diseases (62–64), should be adapted and tested to meet the personal needs of patients with VDB (65).

Interestingly, our study revealed that patients with higher willingness to take risks demonstrated better functioning during their initial visit to the care center. This finding might appear surprising at first sight, considering that higher willingness to take risks is generally associated with a less healthy lifestyle (19, 21). However, in the case of VDB, patients with higher risk-taking tendencies may have developed strategies that contribute to maintaining their functioning. Individuals who are more inclined to take risks might more vigorously engage in activities that challenge their balance and mobility, leading to better adaptation and improved functioning. Additionally, they may be less affected by fears and uncertainties associated with the disease (5, 32). It is important to note that the results of this study should not be interpreted as a recommendation to promote risk-taking behavior in general, given the negative side effects of higher risk-taking found in other studies (19, 21). Instead, future research should focus on unraveling the specific strategies employed by patients with higher risk-taking tendencies to promote functioning.

Several limitations of this study have to be considered. First, most of the data gathered within this study relied on self-reported measures, which may be susceptible to potential information bias. Although we cannot exclude the possibility of such bias being present in our data, we want to emphasize that validated instruments were used, wherever available, and data collection and processing was accompanied by constant quality controls. The assessment of the personality traits relied on a set of self-assessment questions, rather than more extensive choice experiments. Usually, in the economic literature risk attitudes are elicited by a series of hypothetical or even monetarily incentivized lotteries, whereas setting to measure time preferences describe intertemporal trade-offs. However, our sample comprised a considerable portion of frail study participants, many of whom were older and in poor health. These participants might have been overwhelmed by the often-demanding choice experiments, resulting in biased, inaccurate, or incomplete data. Preference modules involving such comparably simple questions used within this study are well established and yielded good and comparable results in the past (66). Second, while personality traits had long been considered to be mostly stable over time (67), recent studies have questioned this traditional assumption, especially in the cases of health shocks (68, 69). This might also be of relevance in our field of application. Though it may be possible that self-efficacy, risk attitudes, or time preferences have changed between the baseline assessment and the follow-up, especially in the case of (very) successful or (very) unsuccessful treatments, a follow-up time of 3 months likely was too short for profound changes in the personality structure. Third, the data collection of this study took place during the COVID-19 pandemic, which might have influenced participants' experiences, perceptions, and behaviors, thus potentially entailing systematic differences in their personality traits. These pandemic-related factors could have affected the generalizability of our findings to non-pandemic periods. One indication for such an effect could be a systematic difference in the personality traits between time points of high incidence rates and rigorous restrictions and time points of low incidence rates and more loosened restrictions through the course of the baseline assessment. Though this was not the focus of this article, a performed descriptive sensitivity analysis showed that the personality traits remained somewhat stable over the course of the baseline assessment and did not reveal any indication of temporal trends.

Patients with vertigo, dizziness, or balance disorders often face considerable limitations and restrictions in functioning. In conclusion, our study contributes to the understanding of the influence of selected personality traits on functioning in older patients with VDB. Further research is warranted to elucidate the underlying mechanisms driving the observed associations found in this study. Adaptations to current treatment strategies are necessary to improve functioning as some patient groups, especially those with low self-efficacy, don't seem to benefit from current care pathways. Our findings provide an initial foundation for the development of tailored interventions that address personality traits, thereby contributing to the optimization of VDB management strategies. Promoting self-efficacy through clinical interventions and thoughtful communication can empower patients to play an active part in their treatment and thus holds promise for improving functioning and overall wellbeing in individuals with VDB.

## Data availability statement

The datasets presented in this article are not readily available because of data privacy regulations that apply in the country of data collection. Requests to access the datasets should be directed to BK, benedict.katzenberger@med.uni-muenchen.de.

## Ethics statement

The studies involving humans were approved by the Ethics Committee at the Medical Faculty of Ludwig Maximilian University of Munich (#20-727). The studies were conducted in accordance with the local legislation and institutional requirements. The participants provided their written informed consent to participate in this study.

## Author contributions

BK: Conceptualization, Data curation, Formal analysis, Investigation, Methodology, Project administration, Visualization, Writing—original draft, Writing—review & editing. SF: Methodology, Validation, Writing—review & editing, Data curation. LS: Conceptualization, Funding acquisition, Supervision, Writing—review & editing. RS: Methodology, Validation, Writing—review & editing. AH: Validation, Visualization, Writing—review & editing. DK: Project administration, Writing—review & editing. EG: Conceptualization, Funding acquisition, Resources, Supervision, Writing—review & editing.
