# Supplementary material for: Association of self-efficacy, risk attitudes, and time preferences with functioning in older patients with vertigo, dizziness, and balance disorders in a tertiary care setting—Results from the MobilE-TRA2 cohort
Source: Front Neurol. 2023 Dec 15;14:1316081. doi: 10.3389/fneur.2023.1316081 (PMC10755024; doi:10.3389/fneur.2023.1316081)
Supplement: Supplementary file 1 [file Data_Sheet_1.pdf]

Supplementary Material

Supplemental Figure 1 Directed Acyclic Graph (DAG) used to identify the minimal sufficient adjustment set

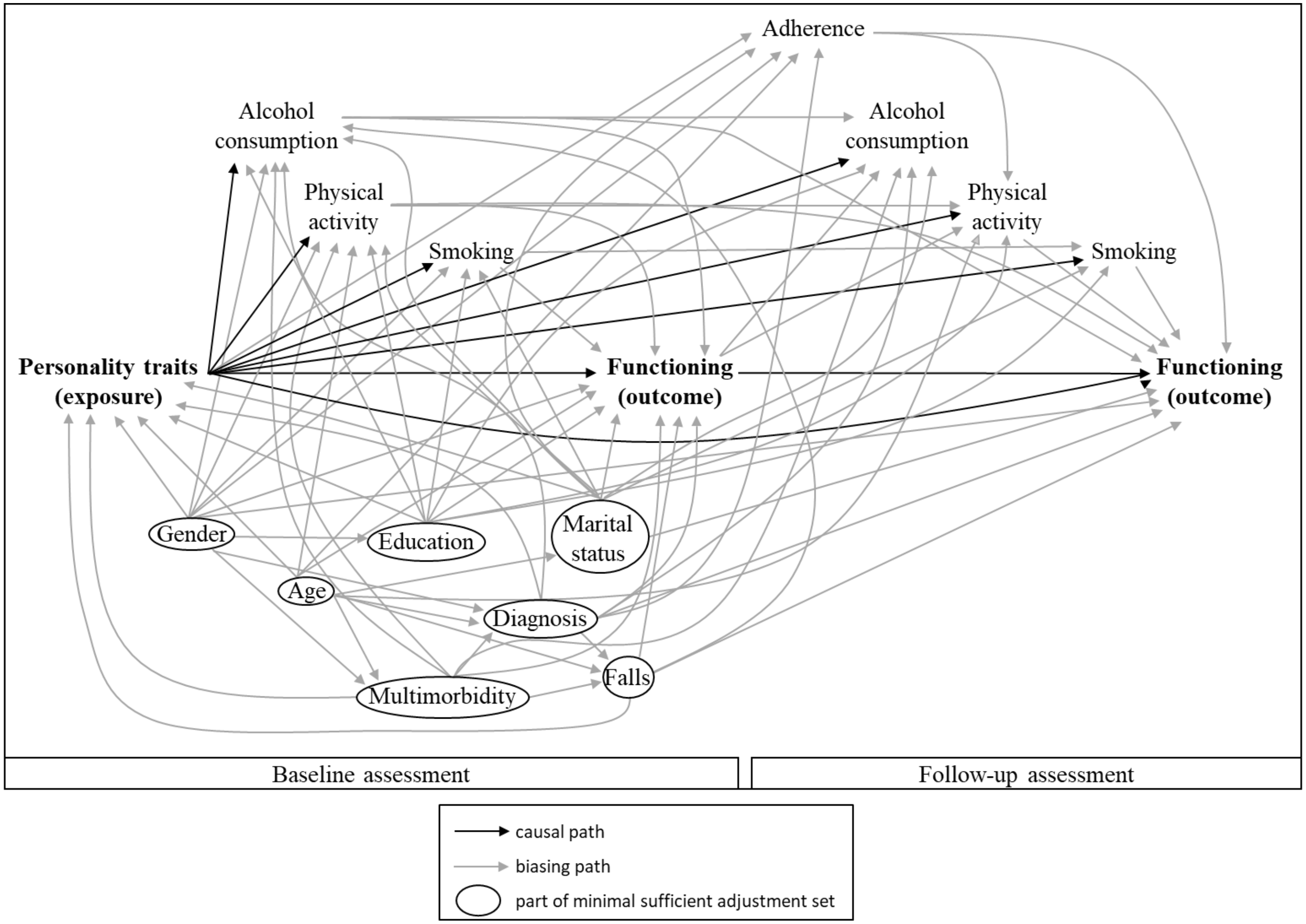

Supplemental Table S1 Assessment of comorbidities in the DizzyReg patient registry

The assessment of the comorbidities was based on patients' self-report during baseline and follow-up assessment. Patients were given the following list of comorbidities:

- Asthma
- Chronic obstructive pulmonary disease or emphysema
- Chronic bronchitis
- Neurological disease (multiple sclerosis, epilepsy)
- Liver disease (e.g. cirrhosis)
- Cardiac insufficiency
- Angina pectoris, coronary sclerosis
- Cardiac arrhythmia
- Hay fever
- Kidney disease
- Inflammatory joint disease
- Hypertension
- Other diseases

Supplemental Table S2 Scores of the Dizziness Handicap Inventory at follow-up stratified by diagnosis (n=229).

	Diagnosis												
	Overall	BPPV	Unilateral vestibulopathy	Bilateral vestibulopathy	Ménière's disease	Vestibular migraine	Central vestibular	Functional vertigo	Orthostatic vertigo	Polyneuropathy	Multifactorial	Other	p-value[a]
N (%)	299	44	16	14	31	29	19	39	25	37	25	20	
**Functioning**													
DHI overall score (mean, SD)	38.56 (22.50)	37.19 (21.03)	36.93 (24.75)	46.43 (21.44)	35.61 (19.96)	32.96 (24.30)	41.89 (27.59)	43.74 (19.42)	28.96 (18.33)	43.72 (21.22)	46.16 (21.57)	29.60 (28.43)	0.037
DHI functional score (mean, SD)	15.49 (10.07)	15.38 (9.27)	14.53 (10.13)	18.57 (9.33)	14.39 (9.34)	12.43 (10.48)	17.67 (12.31)	17.79 (9.24)	11.84 (8.75)	17.06 (9.20)	19.12 (11.03)	11.20 (11.51)	0.047
DHI physical score (mean, SD)	11.33 (7.08)	11.12 (6.71)	12.80 (7.88)	14.29 (7.48)	9.16 (6.59)	8.15 (6.90)	12.67 (7.32)	12.00 (5.68)	8.96 (5.14)	13.56 (7.45)	14.48 (7.51)	8.80 (7.98)	0.003
DHI emotional score (mean, SD)	11.66 (8.18)	10.42 (7.48)	9.60 (8.39)	13.57 (7.57)	12.06 (9.00)	11.86 (9.32)	11.79 (9.91)	13.95 (7.00)	8.16 (7.50)	13.19 (7.29)	12.56 (6.29)	9.60 (10.52)	0.210

BPPV = Benign paroxysmal positional vertigo; DHI = Dizziness Handicap Inventory; SD = Standard deviation
[a] ANOVA

Supplemental Table S3 Longitudinal linear mixed models to assess the association between personality traits and functioning. Higher scores indicate worse functioning. Significant findings are printed in bold.

	Dizziness Handicap Inventory (95% - CI)			
	M1: Overall score	M2: Functional score	M3: Physical score	M4: Emotional score
Observations (n)	550 (301)	552 (301)	553 (302)	554 (302)
(Intercept) [a]	44.87 (33.36 ; 56.39)	18.55 (13.27 ; 23.83)	11.75 (7.92 ; 15.58)	14.65 (10.35 ; 18.94)
Wave [a]				
Baseline	Reference	Reference	Reference	Reference
Follow-up (3 months later)	**-2.56 (-4.49 ; -0.63)**	**-1.18 (-2.04 ; -0.31)**	-0.05 (-0.77 ; 0.68)	**-1.44 (-2.18 ; -0.71)**
**Personality traits (centered to the respective mean)**				
Self-efficacy [b]				
Self-efficacy	**-3.98 (-6.69 ; -1.27)**	**-1.86 (-3.09 ; -0.62)**	-0.56 (-1.47 ; 0.34)	**-1.60 (-2.60 ; -0.60)**
Self-efficacy * time	**-4.35 (-6.68 ; -2.02)**	**-1.69 (-2.73 ; -0.64)**	**-1.10 (-1.96 ; -0.24)**	**-1.48 (-2.36 ; -0.60)**
Health-related risk attitudes [c]				
Risk attitude	**-1.35 (-2.34 ; -0.36)**	**-0.55 (-1.00 ; -0.10)**	-0.07 (-0.41 ; 0.27)	**-0.75 (-1.12 ; -0.38)**
Risk attitude * time	0.28 (-0.57 ; 1.13)	0.16 (-0.22 ; 0.53)	-0.02 (-0.34 ; 0.29)	0.14 (-0.18 ; 0.47)
Time preferences - Present-orientation (PO) [d]				
PO	0.24 (-2.01 ; 2.50)	-0.10 (-1.13 ; 0.92)	0.24 (-0.52 ; 1.01)	0.13 (-0.71 ; 0.97)
PO * time	-0.75 (-2.69 ; 1.19)	-0.28 (-1.14 ; 0.59)	0.04 (-0.68 ; 0.77)	-0.48 (-1.22 ; 0.26)
**Random effects**				
Intercept (SD)	15.45	7.14	4.90	5.70

All models are controlled for the diagnosis, present falls within the last 12 months, multimorbidity, age, gender, education, and marital status. Significant results are highlighted in bold print.

CI = Confidence interval; M1 – M4 = Models 1 to 4, one model per score; SD = Standard deviation

[a] Applies for patients aged 60 with mean self-efficacy of 3.96, mean risk attitude of 4.63, and mean present-orientation of 2.52.

[b] Centered to mean self-efficacy of 3.96.

[c] Centered to mean risk attitude of 4.63.

[d] Centered to mean present-orientation of 2.52.

References utilized to guide the creation of the DAG used within the analysis

Anderson, L.R., and Mellor, J.M. (2008). Predicting health behaviors with an experimental measure of risk preference. J Health Econ 27(5), 1260-1274. doi: 10.1016/j.jhealeco.2008.05.011.

Axon, R.N., Bradford, W.D., and Egan, B.M. (2009). The role of individual time preferences in health behaviors among hypertensive adults: a pilot study. J Am Soc Hypertens 3(1), 35-41. doi: 10.1016/j.jash.2008.08.005.

Bisdorff, A., Von Brevern, M., Lempert, T., and Newman-Toker, D.E. (2009). Classification of vestibular symptoms: towards an international classification of vestibular disorders. J Vestibul Res 19(1-2), 1-13. doi: 10.3233/VES-2009-0343.

Bogliacino, F., Codagnone, C., Montealegre, F., Folkvord, F., Gomez, C., Charris, R., et al. (2021). Negative shocks predict change in cognitive function and preferences: assessing the negative affect and stress hypothesis. Sci Rep 11(1), 3546. doi: 10.1038/s41598-021-83089-0.

Bradford, W.D. (2010). The association between individual time preferences and health maintenance habits. Med Decis Making 30(1), 99-112. doi: 10.1177/0272989X09342276.

Decker, S., and Schmitz, H. (2016). Health shocks and risk aversion. J Health Econ 50, 156-170. doi: 10.1016/j.jhealeco.2016.09.006.

Dohmen, T., Falk, A., Huffman, D., Sunde, U., Schupp, J., and Wagner, G.G. (2011). Individual risk attitudes: Measurement, determinants, and behavioral consequences. J Eur Econ Assoc 9(3), 522-550. doi: 10.1111/j.1542-4774.2011.01015.x.

Dunlap, P.M., Holmberg, J.M., and Whitney, S.L. (2019). Vestibular rehabilitation: advances in peripheral and central vestibular disorders. Curr Opin Neurol 32(1), 137-144. doi: 10.1097/WCO.0000000000000632.

Gast, A., and Mathes, T. (2019). Medication adherence influencing factors-an (updated) overview of systematic reviews. Syst Rev 8(1), 112. doi: 10.1186/s13643-019-1014-8.

Grill, E., Schäffler, F., Huppert, D., Müller, M., Kapfhammer, H.-P., and Brandt, T. (2014). Self-efficacy beliefs are associated with visual height intolerance: a cross-sectional survey. PLoS One 9(12), e116220. doi: 10.1371/journal.pone.0116220.

Holman, H., and Lorig, K. (2014). "Perceived self-efficacy in self-management of chronic disease," in Self-efficacy: Thought control of action, ed. R. Schwarzer. 1 ed (London and New York: Taylor & Francis), 305-324.

Holmes, S., and Padgham, N.D. (2011). A review of the burden of vertigo. J Clin Nurs 20(19-20), 2690-2701. doi: 0.1111/j.1365-2702.2010.03585.x.

Jacobson, G.P., and Newman, C.W. (1990). The development of the dizziness handicap inventory. Arch Otolaryngol 116(4), 424-427. doi: 10.1001/archotol.1990.01870040046011.

Jeng, Y.J., and Young, Y.H. (2020). Evolution of vestibular disorders in older adults: From young-old to middle-old to oldest-old. Geriatr Gerontol Int 20(1), 42-46. doi: 10.1111/ggi.13813.

Lahmann, C., Henningsen, P., Brandt, T., Strupp, M., Jahn, K., Dieterich, M., et al. (2015). Psychiatric comorbidity and psychosocial impairment among patients with vertigo and dizziness. J Neurol Neurosur Ps 86(3), 302-308. doi: 10.1136/jnnp-2014-307601.

Lawless, L., Drichoutis, A.C., and Nayga, R.M. (2013). Time preferences and health behaviour: a review. Agric Econ 1(17), 1-19. doi: 10.1186/2193-7532-1-17.

Madsen, K.P., Kjaer, T., Skinner, T., and Willaing, I. (2019). Time preferences, diabetes self-management behaviours and outcomes: a systematic review. Diabet Med 36(11), 1336-1348. doi: 10.1111/dme.14102.

Monzani, D., Casolari, L., Guidetti, G., and Rigatelli, M. (2001). Psychological distress and disability in patients with vertigo. J Psychosom Res 50(6), 319-323. doi: 10.1016/s0022-3999(01)00208-2.

Mueller, M., Schuster, E., Strobl, R., and Grill, E. (2012). Identification of aspects of functioning, disability and health relevant to patients experiencing vertigo: a qualitative study using the international classification of functioning, disability and health. Health Qual Life Outcomes 10, 1-9. doi: 10.1186/1477-7525-10-75.

Obermann, M., Bock, E., Sabev, N., Lehmann, N., Weber, R., Gerwig, M., et al. (2015). Long-term outcome of vertigo and dizziness associated disorders following treatment in specialized tertiary care: the Dizziness and Vertigo Registry (DiVeR) Study. J Neurol 262(9), 2083-2091. doi: 10.1007/s00415-015-7803-7.

Rueda, S., Artazcoz, L., and Navarro, V. (2008). Health inequalities among the elderly in western Europe. J Epidemiol Commun H 62(6), 492-498. doi: 10.1136/jech.2006.059279.

Ryan, A., Wallace, E., O'Hara, P., and Smith, S.M. (2015). Multimorbidity and functional decline in community-dwelling adults: a systematic review. Health Qual Life Out 13, 1-13. doi: 10.1186/s12955-015-0355-9.

Stel, V.S., Smit, J.H., Pluijm, S.M., and Lips, P. (2004). Consequences of falling in older men and women and risk factors for health service use and functional decline. Age Ageing 33(1), 58-65. doi: 10.1093/ageing/afh028.

Stephan, A.J., Schwettmann, L., Meisinger, C., Ladwig, K.H., Linkohr, B., Thorand, B., et al. (2021). Living longer but less healthy: The female disadvantage in health expectancy. Results from the KORA-Age study. Exp Gerontol 145, 111196. doi: 10.1016/j.exger.2020.111196.

Tschan, R., Best, C., Beutel, M.E., Knebel, A., Wiltink, J., Dieterich, M., et al. (2011). Patients' psychological well-being and resilient coping protect from secondary somatoform vertigo and dizziness (SVD) 1 year after vestibular disease. J Neurol 258(1), 104-112. doi: 10.1007/s00415-010-5697-y.

van der Pol, M. (2011). Health, education and time preference. Health Econ 20(8), 917-929. doi: 10.1002/hec.1655.

Whitney, S.L., Alghadir, A.H., and Anwer, S. (2016). Recent evidence about the effectiveness of vestibular rehabilitation. Curr Treat Option Ne
